# Feeding on resistant rice leads to enhanced expression of defender against apoptotic cell death (*OoDAD1*) in the Asian rice gall midge

**DOI:** 10.1186/s12870-015-0618-y

**Published:** 2015-10-01

**Authors:** Deepak K. Sinha, Isha Atray, JS Bentur, Suresh Nair

**Affiliations:** Plant Molecular Biology Group, International Centre for Genetic Engineering and Biotechnology, Aruna Asaf Ali Marg, New Delhi, 110 067 India; Directorate of Rice Research, Rajendranagar, Hyderabad, 500 030 India; Agri Biotech Foundation, Rajendranagar, Hyderabad, 500 030 India

**Keywords:** Plant-insect interactions, Incompatible interactions, Hypersensitive response, Apoptosis, *Orseolia oryzae*, *Oryza sativa*, Gall formation

## Abstract

**Background:**

The Asian rice gall midge (*Orseolia oryzae*) is a destructive insect pest of rice. Gall midge infestation in rice triggers either compatible or incompatible interactions leading to survival or mortality of the feeding maggots, respectively. In incompatible interactions, generation of plant allelochemicals/defense molecules and/or inability of the maggots to continue feeding on the host initiate(s) apoptosis within the maggots. Unraveling these molecular events, triggered within the maggots as a response to feeding on resistant hosts, will enable us to obtain a better understanding of host resistance. The present study points towards the likely involvement of a defender against apoptotic cell death gene (*DAD1*) in the insect in response to the host defense.

**Results:**

The cDNA coding for the *DAD1* orthologue in the rice gall midge (*OoDAD1*) consisted of 339 nucleotides with one intron of 85 bp and two exons of 208 and 131 nucleotides. The deduced amino acid sequence of *OoDAD1* showed a high degree of homology (94.6 %) with DAD1 orthologue from the Hessian fly (*Mayetiola destructor*) —a major dipteran pest of wheat. Southern hybridization analysis indicated that *OoDAD1* was present as a single copy in the genomes of the Asian rice gall midge biotypes (GMB) 1, 4 and 4 M. In the interactions involving GMB4 with Jaya (susceptible rice host) the expression level of *OoDAD1* in feeding maggots gradually increased to 3-fold at 96hai (hours after infestation) and peaked to 3.5-fold at 96hai when compared to that at 24 hai. In contrast, expression in maggots feeding on RP2068 (resistant host) showed a steep increase of more than 8-fold at 24hai and this level was sustained at 48, 72 and 96hai when compared with the level in maggots feeding on Jaya at 24hai. Recombinant OoDAD1, expressed in *E. coli* cells, when injected into rice seedlings induced a hypersensitive response (HR) in the resistant rice host, RP2068, but not in the susceptible rice variety, Jaya.

**Conclusions:**

The results indicate that the expression of *OoDAD1* is triggered in the feeding maggots probably due to the host resistance response and therefore, is likely an important molecule in the initial stages of the interaction between the midge and its rice host.

**Electronic supplementary material:**

The online version of this article (doi:10.1186/s12870-015-0618-y) contains supplementary material, which is available to authorized users.

## Background

Apoptosis or programmed cell death features stereotypical morphological changes such as shrinking of cell, cell deformation, condensation of chromatin and finally cell fragmentation into apoptotic bodies. These changes are the consequences of several biochemical and molecular events occurring within the cell [1]. Such events are executed and regulated by various molecules within the animal cell [2]. One such regulator, DAD1 (defender against apoptotic cell death) was identified and proved to be interacting with MCL1 (a member of the BCL2 protein family) providing a new perspective on its putative role in apoptosis. The *DAD1* gene was originally isolated during complementation studies of a mutant hamster cell line undergoing apoptosis upon incubation at non-permissive temperatures. Further, these tsBN7 cells could be rescued at the non-permissive temperature upon transfection with the DAD1 wild-type gene [3].

Involvement of *DAD1* in regulation of apoptosis or apoptosis related pathways have since been widely reported. *DAD1* was reported to be involved during development of *C. elegans* embryos [4] and *Bombyx mori* [5]. Involvement of DAD1 in temperature induced apoptotic cell death was reported in *Araneus ventricosus* and *Argopecten irradians* [6, 7]. In the plant kingdom, the role of DAD1 homologues has been reported in *Arabidopsis thaliana* [8], pea [9] and rice [10]. Differential regulation of *DAD1* gene was observed in flower petals during the senescence phase [9]. Results of these investigations indicated the important role played by *DAD1* in apoptosis and development in both animal and plant systems. Up-regulation of *DAD1*-like anti-apoptotic genes was speculated in insect pests upon stress encountered due to plant defense molecules [11]. Interestingly, *DAD1* homologue was up-regulated in one such insect pest, Hessian fly (*Mayetiola destructor*), feeding on resistant wheat (wheat host that initiates hypersensitive mediated defense response upon Hessian fly infestation), suggesting its role in inhibition of unwanted apoptosis triggered due to the defense response of the host [11]. As observed in the Hessian fly-wheat interaction, the Asian rice gall midge (*Orseolia oryzae*) infestation in rice is known to induce a similar type of defense response. However, no reports on the role of any anti-apoptotic genes in the rice-gall midge interaction are available.

The Asian rice gall midge is the third major insect pest of rice. Estimated economic loss incurred upon gall midge attack amounts to $80 million in India alone [12]. More recently, as a result of extensive research towards understanding the galling interaction [13–16] and with the availability of vast amounts of sequence data [16], rice-gall midge is emerging as a useful model system for understanding the molecular and physiological events that enable insects to overcome the host defense machinery. The gall midge-rice interaction is either compatible or incompatible [17]. During compatible interaction, the gall midge manipulates the host to survive and induces gall formation; whereas during incompatible interaction the host defense overcomes the strategies adopted by the insect leading to the mortality of the gall midge. Incompatible interaction can be classified into two types: HR+ and HR-. HR+ type interaction is manifested by a hypersensitive response and cell death in the plant at the region of entry of the gall midge maggots whereas HR-type interaction is non-hypersensitive mediated defense response. Inability of the insect maggots to feed due to production of plant allelochemicals/defense molecules results in the death of the maggots in an incompatible interaction and the maggots usually die within 96 h post egg hatching [18].

Various studies have described the hypersensitive mechanism in the host plants [19]. However, there are few studies that investigated and compared the response of the insect during hypersensitive incompatible or compatible interaction. This work was initiated with the hypothesis that the maggots feeding on resistant rice plants encounter greater stress-induced challenge when compared to maggots feeding on susceptible rice plants. This stress encountered by the insect in the resistant host, generated by the plant defense molecules [20] and the inability to feed thereafter, leads to initiation of apoptosis within the insect. In order to survive, the likely survival mechanism in the insect would be the up-regulation of anti-apoptotic genes [11]. *DAD1* is an important apoptotic suppressor gene and till date there has been no report on *DAD1*-like genes from the Asian rice gall midge. Also, it is still unclear, if secretions from the gall midge feeding on the rice pant trigger apoptosis in the host plant or not. Therefore, we deemed it pertinent to clone, characterize and express *DAD1* from the gall midge and evaluate its role in the insect and corresponding host responses, if any.

The current study describes characterization of *DAD1* from the Asian rice gall midge and its transcriptional expression patterns in the insect during compatible and incompatible interactions with its host. *Orseolia oryzae DAD1* (*OoDAD1*) was isolated from a cDNA library generated from maggots feeding on susceptible and resistant host varieties [21]. Transcriptional over-expression of *OoDAD1* observed in maggots during compatible and incompatible interactions suggested its role in regulation of unwanted apoptosis. In addition, this study for the first time demonstrated that the host plant recognizes DAD1 from the insect, leading to the induction of a hypersensitive mediated response. Results of the present investigation revealed the important role played by DAD1 in insect-plant interaction.

## Results

### Characterization of OoDAD1

The full-length cDNA of *OoDAD1* consisted of 339 nucleotide bases [GenBank:KP890835] coding for 113 amino acids with a predicted molecular mass of 12.7 kDa. The cDNA clone was designated *OoDAD1* and the genomic sequence was designated *gOoDAD1* [GenBank:KP890834]. The latter consisted of one intron of 85 bp and two exons of 208 and 131 nucleotides.

The estimated pI of the predicted protein OoDAD1 was found to be 9.18. There were eight non-polar and seven polar amino acid residues. The instability index, as computed by ExPASy-ProtParam tool, was 36.33 that classified the protein as a stable protein. TOPCONS predicted OoDAD1 to possess three trans-membrane helices (Additional file 1: Figure S1). Bioinformatics analysis using SMART predicted the absence of a secretory signal sequence in the predicted protein while TOPCONS confirmed that the protein was likely to be localized to membranes. Maximum homology of the deduced amino acid sequence was observed with DAD1 from *Mayetiola destructor* (MdesDAD1; 89 %, 3e–52; Acc. No. ABY21317) DAD1 followed by DAD1 from *Anopheles gambiae* (78 %, 2e–47; Acc. No. AAQ94040). Secondary structure prediction tool, ROBETTA, predicted 5 probable structures for OoDAD1 (Additional file 2: Figure S2). Of all the predicted models the fifth model was found to have lowest score (lowest energy) and the maximum stability. OoDAD1 was predicted to possess four large alpha-helices, as reported in MdesDAD1, with no beta strands across the entire deduced protein sequence.

**Fig. 1:**
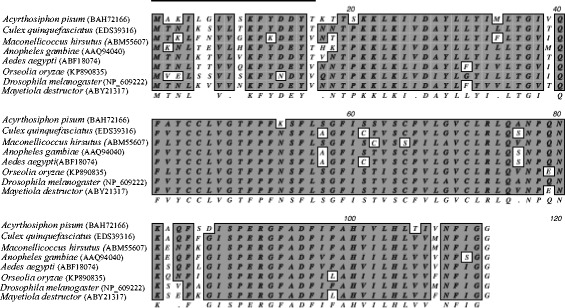
Multiple sequence alignment of OoDAD1 with its orthologues from blood-feeding, sap-sucking and phytophagous insects. Black line indicates the variable N-terminal region of the proteins compared. Shaded boxes highlight conserved domains. Accession numbers are in parenthesis. Conserved amino acid residues are shown below the shaded boxes

A multiple sequence alignment of the predicted amino acid sequence of DAD1 (Fig. 1) from different insects with OoDAD1 revealed several homologous domains and conserved regions. Results indicated that the protein was highly conserved at the C-terminal region as compared to the N-terminus. The highest degree of homology (similarity score) was 94.6 % in case of *Mayetiola destructor* (Acc. No. ABY21317).

### Phylogenetic analysis

**Fig. 2:**
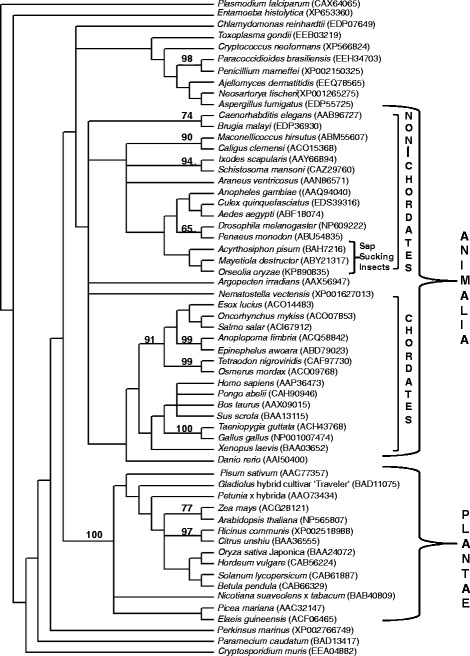
Phylogenetic tree showing relationship between orthologues of DAD1 reported from different organisms. The tree was constructed using the Neighbour-joining method and the pair-wise distances were calculated using the Poisson-corrected distance method included in the MacVector suite of programs. Branch lengths are arbitrary. This tree was arrived at using 1000 replications and figures at nodes represent detected bootstrap values above 50 %. Accession numbers are in parenthesis

Phylogenetic analysis of OoDAD1 revealed the degree of relationship of OoDAD1 with respect to those from other organisms (Fig. 2). However, this study clearly classified DAD1 of plant and animal taxa into two large clades. The clade containing DAD1 from animals were further sub-divided into vertebrates and invertebrates. The dipterans were clustered in a sub-clade under the invertebrates group. OoDAD1 was grouped within the clade containing other insects belonging to the order Diptera. The tree also revealed close relationship of OoDAD1 with orthologues from the Hessian fly (*Mayetiola destructor*) and aphid (*Acrythosiphon pisum*) both of which are also plant feeders.

### Southern analysis

**Fig. 3:**
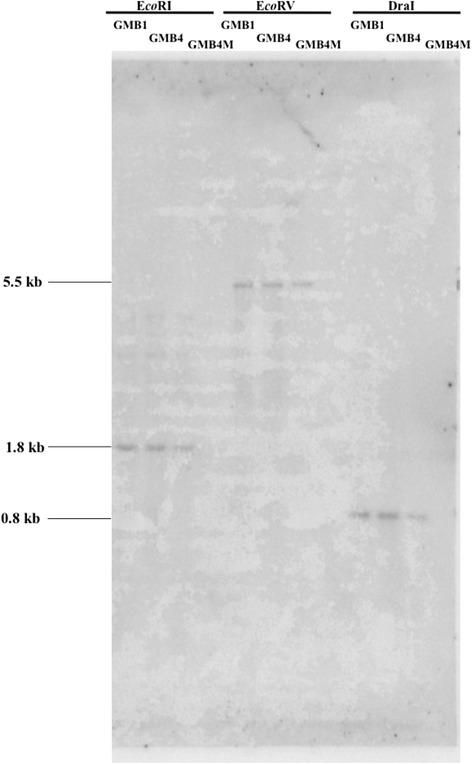
Southern analysis of genomic DNAs of Asian rice gall midge biotypes (GMB1, GMB4 and GMB4M). The DNAs were digested with *Eco*RI, *Eco*RV and *Dra*I and probed with a 339 bp fragment of *OoDAD1*. Molecular mass (in kb) of the hybridization signals is indicated by figures on the left

Southern hybridization, using a 339 bp *OoDAD1* fragment as probe, revealed that *OoDAD1* existed as a single copy in all the three biotypes (GMB1, GMB4 and GMB4M) analysed (Fig. 3). A single hybridization signal was observed in all the three biotypes digested with restriction enzymes *Eco*RI, *Eco*RV and *Dra*I. Further, no restriction fragment length polymorphism (RFLP) was detected between these biotypes.

### Differential expression analysis of OoDAD1 in maggots feeding on susceptible and resistant rice varieties

**Fig. 4:**
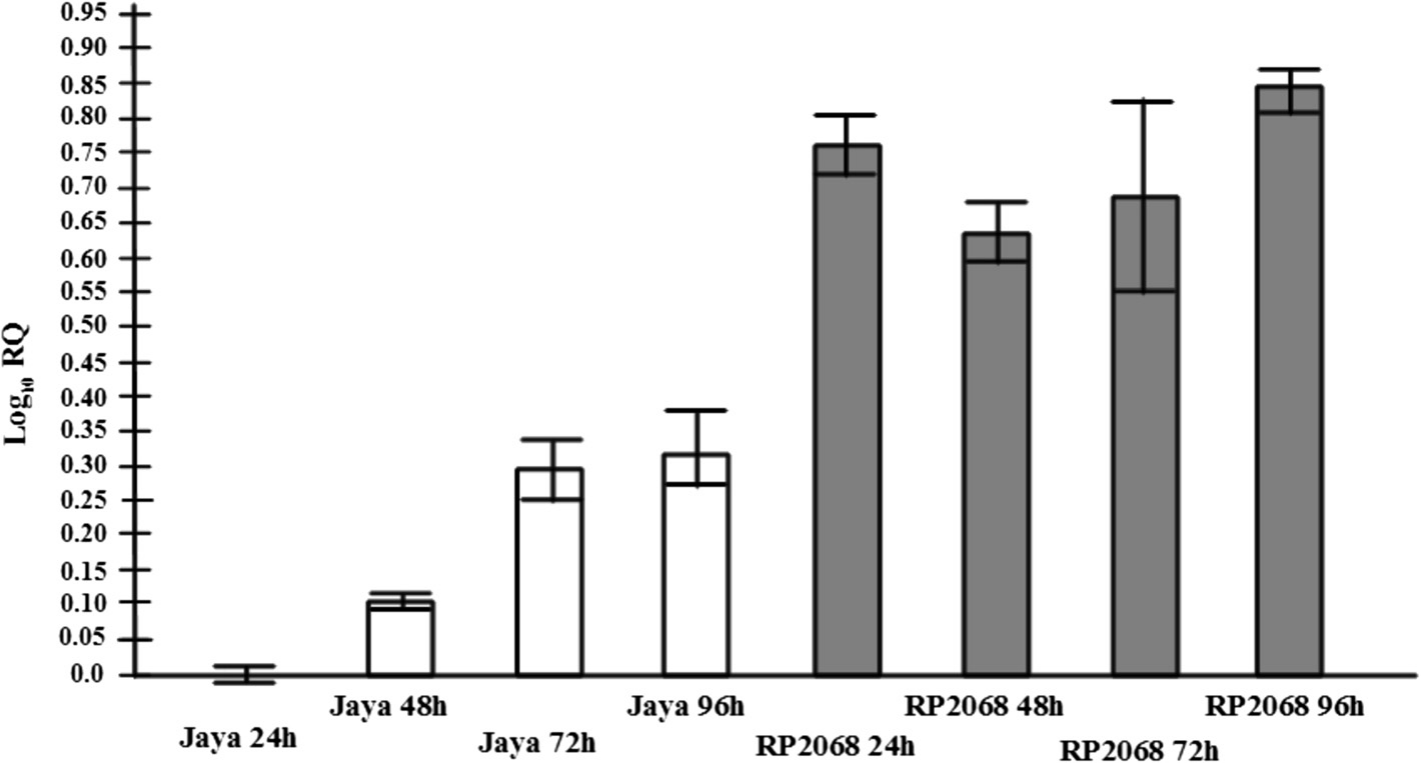
Expression of *OoDAD1* in the Asian rice gall midge. Relative expression evaluated in midges feeding on susceptible [Jaya (white bars)] and resistant [RP2068 (shaded bars)] rice varieties determined using Quantitative Real-Time PCR. The time points mentioned are 24, 48, 72 and 96hai. RQ values describe the relative expression values of transcripts with reference to expression level of *OoDAD1* in maggots feeding on Jaya variety (24hai). Error bars represent mean ± SD

The transcript level of *OoDAD1* was assessed in a set of compatible (Jaya-GMB4) and incompatible interactions (RP2068-GMB4) (Fig. 4). The expression level of *OoDAD1* transcripts at 24 h in maggots feeding on the susceptible (Jaya) host was used as a baseline and the expression at all other time points were scored relative to this. In the interactions involving GMB4 with Jaya (susceptible host) and RP2068 (resistant host), over-expression of *OoDAD1* was observed in both cases. However, the expression level in maggots feeding on Jaya gradually increased by more than 3-fold at 72hai and peaked to 3.5-fold at 96hai [0.35 (log10) fold] (hours after infestation) in comparison to maggots feeding on Jaya 24hai. However, in case of maggots feeding on RP2068 there was sudden increase of expression level to 8-fold from 24hai [0.8 (log10) fold] and this enhanced expression was sustained at 48hai [0.65 (log10) fold], 72hai [0.70 (log10) fold] and 96hai [0.85 (log10) fold].

### Generation of recombinant OoDAD1

**Fig. 5:**
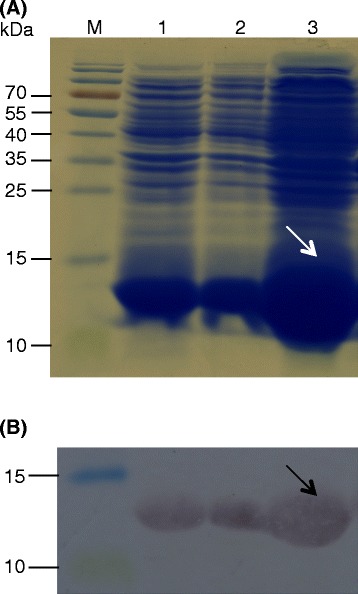
Heterologous expression of OoDAD1 and Western analysis. **a** Coomassie blue stained sodium dodecyl sulphate polyacrylamide gel showing over-expression of recombinant OoDAD1 in BL21 (DE3) pLysE *Escherichia coli*-based expression system using pET 28a expression vector. Lanes: 1, lysate of induced, transformed cells with *OoDAD1*; 2, supernatent of induced, transformed cells with *OoDAD1*; 3, pellet of induced, transformed cells with *OoDAD1*. M, protein ladder. **b** Western analysis of polyacrylamide gel shown in (**a**) and electrotransferred to a nitrocellulose membrane and probed with anti-His tag antibodies (see Experimental Procedures). Lane designation is same as in (**a**). Arrows indicate the location of the His-tagged OoDAD1. Numbers on the left represent molecular weights in kilodaltons (kDa)

The use of pET 28a vector for protein expression resulted in the production of His-tagged OoDAD1 protein with a molecular weight of 13.2 kDa, (including the seven His-residues in the N-Terminal region) as expected. Anti-His antibodies were used to confirm the expression and the size of the protein (Fig. 5).

### Plant assay and DAB (3,3′-diaminobenzidine) staining

**Fig. 6:**
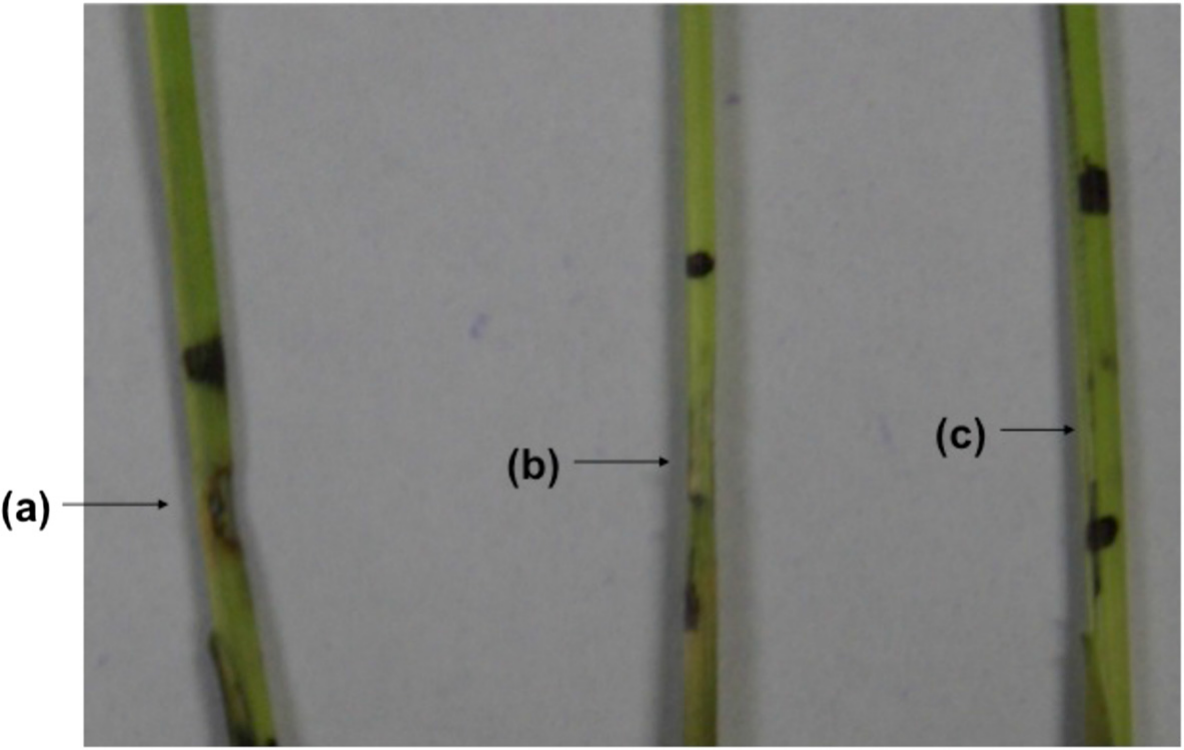
HR response of gall midge resistant rice injected with recombinant OoDAD1. Plant injection assay showing induction of HR response in the gall midge resistant rice variety, RP2068, upon injection with recombinant OoDAD1. RP2068 injected with **a** purified protein; **b** protein elution buffer; **c** BSA dissolved in protein elution buffer. Black dots indicate the markings made prior to injection for easy localization of injected regions

**Fig. 7:**
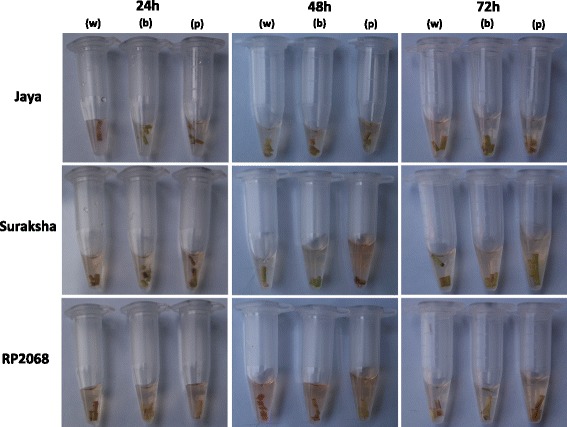
DAB staining confirms HR response in resistant rice varieties injected with OoDAD1. DAB (3,3′-diaminobenzidine)-staining of recombinant OoDAD1-injected rice plants to show HR reaction in gall midge resistant rice varieties (Suraksha and RP2068) compared with the gall midge susceptible variety, Jaya, at 24, 48 and 72hai post-injection with water (w), buffer (b) and recombinant OoDAD1 (p) (see Experimental Procedures). Two of the resistant rice varieties (Suraksha and RP2068) showed HR (browning of DAB-stained tissue)

The purified OoDAD1 protein (purified using Ni-NTA column), the protein elution buffer and water injection into the host variety RP2068, initiated HR. However, the observed spread of HR in RP2068 (Fig.6a) was more in plants injected with OoDAD1 when compared with the plants injected with buffer and BSA. However, no HR was observed in Jaya plants after injection (Additional file 3: Figure S3). The injection region of the plants when stained with DAB showed brown coloration in Suraksha after 48 h post injection and after 72 h post injection in RP2068 (Fig. 7). DAB staining produces brown coloration in tissues having increased peroxidase activity (increased production of reactive oxygen species) and is used as a marker for hypersensitivity in plants.

## Discussion

For a better understanding of the molecular basis of insect-plant interaction, studying the defense response initiated by the avirulent maggots, feeding on a resistant host, is equally important as gaining insight into the mechanism of infestation of virulent maggots. Initiation of hypersensitive response as a result of apoptosis in the plants has been well documented in case of plant-microbe interaction [22]. However, other than the study on Hessian fly-wheat interaction, there are no additional reports that delve into the modulation of genes related to apoptosis in the insect during its interaction with the host plant [11].

The current study indicated that *OoDAD1* is transcriptionally modulated in the pest depending on whether it is feeding on a susceptible or resistant host. In addition, this is the first report of the heterologous expression of a *DAD1* from an insect which when injected into resistant rice hosts induced a hypersensitive response.

BLAST and phylogenetic analyses not only showed high homology between *DAD1* orthologues of insects and *OoDAD1* but also a high degree of conservation of the predicted amino acid residues of OoDAD1 with those reported for DAD1 from other species including plants and vertebrates. Such a high degree of conservation is likely indicative of an important functional role played by DAD1 in vertebrates, invertebrates and plants. Earlier reports confirm DAD1 to be a member of the oligosaccharyl transferase complex responsible for N-linked glycosylation [23]. It has also been observed that the C-terminal amino acid residues, which are known to be crucial for N-terminal glycosylation function [24], are conserved in DAD1 from a wide range of organisms.

However, despite a high degree of homology between DAD1 from different organisms, a few dissimilar amino acid residues amongst the proteins provide each with specific signatures that can be used to classify DAD1 of different organisms corresponding with their evolutionary relationship. Moreover, these specific signatures are capable of differentiating DAD1 of plants from those in the animal kingdom. In addition, these signatures also differentiate DAD1 from invertebrates and vertebrates and also those from phytophagous and haematophagous dipterans.

DAD1 has been shown to play an important role as a suppressor of the apoptotic pathway in many organisms [4]. Hence, the differential expression pattern of *OoDAD1* in maggots feeding on susceptible and resistant hosts is likely indicative of its important role in the apoptotic pathway in the rice gall midge too. While controlled apoptosis is induced in the cell during developmental stages, apoptosis can also occur when cell is under stress [2]. In the case of gall midge-rice compatible interaction, the midge undergoes its normal life cycle. On susceptible plants, maggots establish a feeding site between 12 and 24 hai and genes involved in growth and development are up-regulated, as also observed in the Hessian fly-wheat interaction [25–27]. And therefore, the minor increase in the *OoDAD1* transcript observed in maggots feeding on susceptible host during 48 to 96hai as compared to 24hai could be a part of normal homeostasis and development [28] of maggots.

However, on the resistant plants, the transcript levels of *OoDAD1* registered an instant increase and these levels were sustained till 96hai. In previous studies involving Hessian fly-wheat interactions, it was observed that Hessian fly maggots failed to establish a feeding site on resistant wheat varieties, and as a result stress responsive genes and those involved in disruption of homeostasis were up-regulated [20, 29]. Further, it is likely that on resistant hosts the maggots face nutritional stress owing to its inability to maintain feeding, probably due to toxic plant compounds and/or feeding deterrents, and as a result face starvation and eventual death. This could result in a cascade of events connected with the defense pathways of which one could be over-expression of DAD1 to prevent it from succumbing to stress-induced apoptosis.

Interestingly, results from the plant injection assays, using recombinant OoDAD1, showed increased HR in gall midge resistant rice hosts which contradicts the reported role of DAD1. A plausible explanation for the observed results could be the presence of certain motifs in the insect-derived DAD1 that the gall midge resistant host recognizes and as a result initiates the hypersensitive reaction against the foreign protein. This is also supported by data from the phylogenetic analysis that clearly differentiates DAD1 of plant and animal origin. Moreover, it has also been suggested that DAD1 (a homologue of Ost2) of one organism may not proceed with the conserved mechanism of PCD signaling pathway in another organism [30]. Furthermore, staining of rice tissues with DAB, after injection of recombinant OoDAD1, showed increased HR in Suraksha at 48hai while in RP2068 it was observed at 72hai. This may be due to inherent genotypic differences in the two resistant rice varieties. Our earlier studies have also shown that these two resistant rice varieties carry gall midge resistance genes *Gm11* and *gm3*, respectively. Though both rice varieties behave differently upon gall midge attack the final outcome in both cases is mortality of the maggots [18, 21].

## Conclusions

In conclusion, the experiments described here show the crucial role played by *OoDAD1* in gall midge-rice interaction. *OoDAD1* is transcriptionally up-regulated in maggots, feeding on resistant host, in an attempt to overcome the challenge faced by the maggots in the resistant host. In addition, plant assays involving recombinant OoDAD1 suggested that the insect protein is detected by the resistant host resulting in HR. However, more detailed studies would be required for further unraveling the role played by OoDAD1 in the gall midge-rice interaction. In addition, RNAi-based studies in conjunction with studies on OoDAD1 mutants would help in understanding the role of DAD1 in insect-plant interaction in general and gall midge-rice interaction in particular. Besides, immuno-localization studies with OoDAD1 would help in furthering our understanding of the molecular events during insect-plant interactions.

### Methods

#### Insect material and DNA extraction

The experimental material consisted of Asian rice gall midge biotype 4 (GMB4) insects maintained in greenhouse [31] at the Directorate of Rice Research, Hyderabad, India. Adult gall midges were collected initially from different gall midge infested areas in India. These insects were reared on Jaya and checked on RP2068 varieties of rice under standard conditions [31]. GMB4 is virulent (forms galls on the host plant) on Jaya (lacks gall midge resistance genes) and avirulent (unable to form galls on the host plant leading to subsequent mortality of the maggots) on RP2068 (possesses gall midge resistance genes) rice variety. DNA was extracted from adult gall midges using micro pestle, which had been chilled in liquid nitrogen. The ground tissue was suspended in extraction buffer (1 % SDS, 0.05 M NaCl; 0.05 M Tris–HCl, pH 8.0; 0.025 M EDTA), followed by Proteinase K and RNase treatment, and purified with phenol: chloroform: isoamyl alcohol (25:24:1), and then with chloroform: isoamyl alcohol (24:1) [32]. The purified genomic DNA was then ethanol precipitated and re-suspended in distilled water.

#### Insect stages, dissections and collection of maggots

In order to identify differentially expressed genes maggots were dissected out from the host at different time intervals of 24, 48, 72 and 96hai. To determine the time intervals, rice seedlings (15-day-old) of both Jaya and RP2068 variety were infested with GMB4 and regularly monitored. The maggots take 4–6 h to reach the apical meristem after formation of eyespot. Individual rice seedlings were dissected under the microscope and the maggots were collected in RNAlater (Ambion, Austin, TX, USA) and stored at–80C till further use. Approximately, 600 maggots per rice variety were dissected out.

#### RNA isolation and genomic clone recovery

RNA was isolated using RNeasy Plus Micro Kit (Qiagen, GmbH, Hilden, Germany) following the manufacturer’s protocol. Two biological replicates, that were temporally separated, were included in this study. First strand cDNA synthesis was performed using Superscript III RT enzyme (Invitrogen, Carlsbad, CA, USA) according to the manufacturer’s protocol. A cDNA library was prepared as mentioned in Sinha et al., 2011 [21]. Genome walking and RACE were performed using the published protocol [33]. Details of primers used in this study are provided in Table 1. PCR products were cloned in Topo TA cloning Vector (Invitrogen, Carlsbad, CA, USA) and sequenced by M/s Macrogen Inc., Seoul, South Korea.

**Table 1:** List of primers used for cloning and quantitative Real Time assays of *OoDAD1*. The ‘Prot’ primers were used for cloning *OoDAD1* in pET 28a expression vector

S.No	Name	Primer sequence (5′➔ 3′)	Annealing Temp. (°C)	Amplicon size (bp)
1	OoDAD-F	ATGACGAATCTAACTACAGTTGTTC	55	339^a^
2	OoDAD-R	CTAACCGATGAAATTGAATACG		424^b^
3	RTDAD-F	CATTGTGCTTACCGGTGTCATT	60	177
4	RTDAD-R	CGTTCCGGTGAGATTCCAAT		
5	RTActin-F	TGAGACACCATCACCGGAATC	60	149
6	RTActin-R	ATCCAAAGGCCAATCGTGAA		
7	ProtDAD-F	GAGACATATGACGAATCTAACTACAGTTGTTC	55	339
8	ProtDAD-R	GAGACTCGAGCTAACCGATGAAATTGAATACG		

^a^Amplicon size when cDNA was used as template

^b^Amplicon size when genomic DNA was used as template

#### Sequence and phylogenetic analysis

Sequence assembly was carried out using Phred and Phrap included in the MacVector suite of programs (MacVector Inc., Cary NC, USA; V: 12.0.5). Sequence similarity and annotations were performed using web-based BLAST programs on the National Centre for Biotechnology Information (NCBI; http://www.ncbi.nlm.nih.gov/) servers. Secretion signal peptide analysis of the predicted amino acid sequence was performed using SMART software (http://smart.embl-heidelberg.de). Molecular weight and pI was calculated using ExPASy-ProtParam tool (http://web.expasy.org/protparam/). PSORT II analysis (Prediction of Protein Sorting Signals and Localization Sites in Amino Acid Sequences, http://psort.hgc.jp/form2.html) was used for identifying the localization sites. In the absence of any matching models, structures were predicted using Rosetta fragment insertion method. The de novo protein threading program ROBETTA (http://robetta.bakerlab.org) was used to predict the secondary structure of OoDAD1.

A phylogenetic tree was constructed using the predicted amino acid sequence of OoDAD1 and reported homologues in other organisms. The homologues of DAD1 from vertebrates, invertebrates and plants were used for this analysis. The tree was constructed using the inbuilt distance/neighbor-joining method provided in the MacVector suite of programs. 1000 replications were used to obtain bootstrap values for the branches. The evolutionary distances were computed using Poisson-corrected distances and the gaps were distributed proportionally.

#### Southern blot analysis

Restriction digestion was performed using genomic DNA (3 μg) isolated from three biotypes using *Eco*R1, *Eco*RV and *Dra*1 restriction enzymes (New England Biolabs, Beverly, MA, USA). The digested DNA fragments were electrophoresed on 0.8 % agarose gel (30 V for 12 h) and blotted onto a nylon membrane (GeneScreen Plus, Perkin Elmer, Boston, MA, USA). Transfer of DNA was done using the alkali transfer procedure and the blot was probed with the 339 bp *OoDAD1* fragment cloned from GMB4. The fragment was labeled with α–^32^P deoxycytidine triphosphate using a Nick translation kit (Invitrogen, Carlsbad, CA, USA). Hybridization with the probe and washing of the blot were followed as described by Mohan et al., 1994 [34].

#### Real-Time PCR and statistical analyses

Real Time expression profile was performed for *OoDAD1* gene during different stages of insects feeding on susceptible (Jaya) and resistant (RP2068) hosts. Primer Express (version 3.0; Applied Biosystems, Foster City, CA, USA) was used to design the Real-Time PCR primers for *OoDAD1* and control genes (Table 1). Equal quantity of total RNA (20 ng; as estimated by NanoVue spectrophotometer [GE Healthcare, Little Chalfont, UK]), from different stages were reverse transcribed using Superscript III RT enzyme (Invitrogen, Carlsbad, CA, USA) and oligo (dT) primers according to the manufacturer’s protocol. Amplification efficiency of the designed primers was checked using serially diluted cDNA samples. Actin gene was selected as the internal control after evaluating several candidates using GENORM [35].

The cycling conditions used for Real-Time PCRs were 95 °C for 10 min followed by 40 cycles of 95 °C for 15 s and 60 °C for 1 min. Real-Time PCR was performed using SYBR green chemistry and in Applied Biosystems StepOne Real-Time PCR system. Real-Time PCR mix (20 μl) contained 1XPower SYBR Green PCR mix (Applied Biosystems) and 0.5 mM of the primers. Real-Time PCR was followed by melt curve analysis in order to identify primer dimers and contamination. The amplified fragments were cloned in pCR4-TOPO-TA vector (Invitrogen, Carlsbad, CA, USA) and sequenced to confirm their identity. The quantification of mRNA of OoDAD1 in the maggots was estimated using Relative Standard Curve method. Output data were analyzed using the 2^-ΔΔCt^method, inbuilt into the StepOne real-time PCR analysis software (Applied Biosystems) and results displayed as Relative Expression Values (REVs). Statistical significance of the difference in OoDAD1 expressions between different samples was determined using Student’s *t*-test analysis [36].

#### Heterologous expression of OoDAD1 protein and Western blot analysis

Bacterial expression vector pET 28a (Novagen, Darmstadt, Germany) and BL21 (DE3) pLysE *E.coli* competent cells were used for the production of recombinant OoDAD1. The full-length cDNA, coding for OoDAD1, was PCR amplified using forward and reverse primers (Table 1). The primers were designed with *BamH*1 and *Xho*I restriction sites to enable the cloning of the PCR fragment into their corresponding sites in the pET 28a vector. Chemically competent BL21 (DE3) pLysE *E. coli* were transformed with the designed expression plasmid. LB medium (10 ml) containing 50 μg/ml kanamycin was inoculated with overnight culture (1 %) of transformed cells. LB medium was incubated at 37 °C till the optical density (OD) of the medium reached to 0.6 (OD_600_). 0.5 mM isopropyl-b-D-thiogalactoside (IPTG; Merck, Darmstadt, Germany) was used to induce expression of the His-tagged OoDAD1 protein. The cells were pelleted by centrifugation at 10,000 g for 10 mins after 4 h of induction. The resultant pellet was re-suspended in lysis buffer [25 mM Tris, 10 Mm NaCl, 10 mM benzamidine, 15 % glycerol, 1 mM phenylmethylsulfonyl fluoride (PMSF), 2 mg/ml lysozyme] and centrifuged at 13 000 g for 15 min. The pellet was re-suspended in 1X SDS PAGE dye (50 mM Tris-Cl pH 6.8; 10 % glycerol; 2 % SDS; 12.5 mM ethylenediaminetetraacetic acid; 1 % b-mercaptoethanol; 0.02 % bromophenol blue) and boiled for 5 mins. The sample was electrophoresed in a 15 % SDS polyacrylamide gel and the gel was stained with Coomassie blue dye to visualize the protein bands.

After electrophoresis, the proteins on the gel were electrotransferred onto an Amersham Hybond-ECL nitrocellulose membrane (0.45 μm; GE Healthcare) (100 V for 1 h;using a Mini Trans-Blot cell [Bio-Rad Laboratories, Hercules, CA, USA]). The membrane was blocked with 3 % bovine serum albumin (BSA) dissolved in phosphate-buffered saline (PBS). It was further incubated for 1 h with 1:3000 dilution of 6X His-antibody conjugated to alkaline phosphatase (catalogue no. A7058-1VL; Sigma Aldrich GmbH, Munich, Germany). After repeated washing (3 times) of the membrane with PBS containing 0.05 % Tween-20, His tagged-OoDAD1 was detected using 5-bromo-4-chloro-3′-indolyphosphate/nitro-blue tetrazolium (Sigma Aldrich, St Louis, MO, USA) as substrate. Size estimation of the developed bands was done using a pre-stained protein ladder (Fermentas, Hanover, MD, USA; catalogue # SM0671).

#### Plant injection assay and staining

Fifteen-day-old plants of Jaya, Suraksha and RP2068 were injected with 10 μl (700 ng/μl) of purified recombinant OoDAD1 protein, elution buffer or heat-denatured OoDAD1. Injection of protein was performed using a very fine needle (26G; 0.45 × 13 mm) into the stem of the rice plant carefully. The region to be injected was marked before the injection of the protein in order to localize the injected area. Ten plants per rice variety per time point (24, 48, 72 and 96 h) in three biological replicates were used for this experiment. The injected portion was sliced off after 24, 48, 72 and 96 h post-injection and stained with DAB (3,3′-diaminobenzidine) staining solution as described earlier [37] with minor modifications.

### Availability of supporting data

The nucleotide sequences of full-length *OoDAD1* have been submitted to GenBank under accession numbers KP890835 (cDNA clone) and KP890834 (genomic clone). Other data related to this article are included within the article and its additional files.

## Additional files

Additional file 1: Figure S1. Consensus prediction of membrane protein topology using TOPCONS server indicated the presence of three trans-membrane helices (grey and white boxes in the graph) in the predicted amino acid sequence of OoDAD1. TOPCONS predicted the topology of OoDAD1 from five different topology prediction algorithms: SCAMPI (single sequence mode), SCAMPI (multiple sequence mode), PRODIV-TMHMM, PRO-TMHMM and OCTOPUS. The output of these five algorithms were used as input for the TOPCONS Hidden Markov Model (HMM) (shown in maroon), which provided a consensus prediction for the protein together with a reliability score based on the agreement of the included methods across the sequence. In addition, ZPRED was used to predict the Z-coordinate (*i.e.,* the distance to the membrane center) of each amino acid, and the G-scale was used to predict the free energy of membrane insertion for a window of 21 amino acids centered around each position in the sequence. (PDF 81 kb) Additional file 2: Figure S2. Protein structure of OoDAD1 as predicted by ROBETTA, a *de novo* protein-threading program (http://robetta.bakerlab.org). A, B, C, D and E are the five predicted models and the fifth model was predicted to have lowest score (lowest energy) and therefore, considered the most stable. (PDF 166 kb) Additional file 3: Figure S3. HR response of gall midge resistant rice injected with recombinant OoDAD1. Plant injection assay showing induction of HR response in the gall midge resistant rice variety, Jaya, upon injection with recombinant OoDAD1. Jaya injected with (a) purified protein; (b) protein elution buffer; (c) BSA dissolved in protein elution buffer. Black dots indicate the markings made prior to injection for easy localization of injected regions. (PDF 199 kb)

## Abbreviations

Hai: Hours after infestation
DAB: 3′3′ diaminobenzidine
