# Supplementary material for: Feeding on resistant rice leads to enhanced expression of defender against apoptotic cell death (OoDAD1) in the Asian rice gall midge
Source: BMC Plant Biol. 2015 Oct 1;15:235. doi: 10.1186/s12870-015-0618-y (PMC4591563; doi:10.1186/s12870-015-0618-y)
Supplement: Additional file 1: Figure S1. — Consensus prediction of membrane protein topology using TOPCONS server indicated the presence of three trans-membrane helices (grey and white boxes in the graph) in the predicted amino acid sequence of OoDAD1. TOPCONS predicted the topology of OoDAD1 from five different topology prediction algorithms: SCAMPI (single sequence mode), SCAMPI (multiple sequence mode), PRODIV-TMHMM, PRO-TMHMM and OCTOPUS. The output of these five algorithms were used as input for the TOPCONS Hidden Markov Model (HMM) (shown in maroon), which provided a consensus prediction for the protein together with a reliability score based on the agreement of the included methods across the sequence. In addition, ZPRED was used to predict the Z-coordinate (i.e., the distance to the membrane center) of each amino acid, and the G-scale was used to predict the free energy of membrane insertion for a window of 21 amino acids centered around each position in the sequence. (PDF 81 kb) [file 12870_2015_618_MOESM1_ESM.pdf]

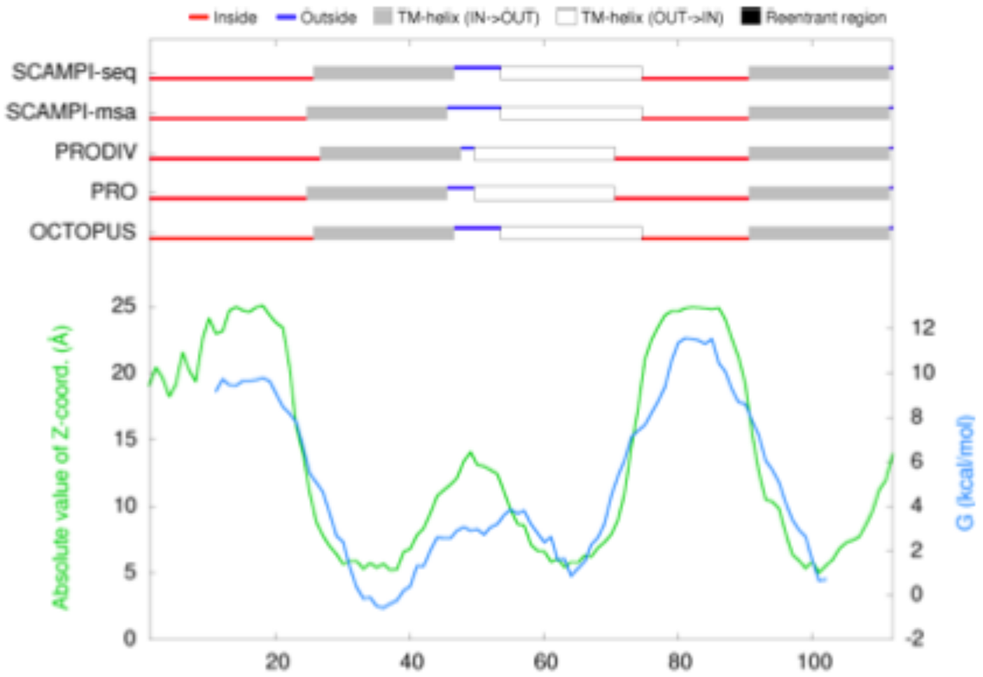

High-resolution image

Consensus prediction (TOPCONS):

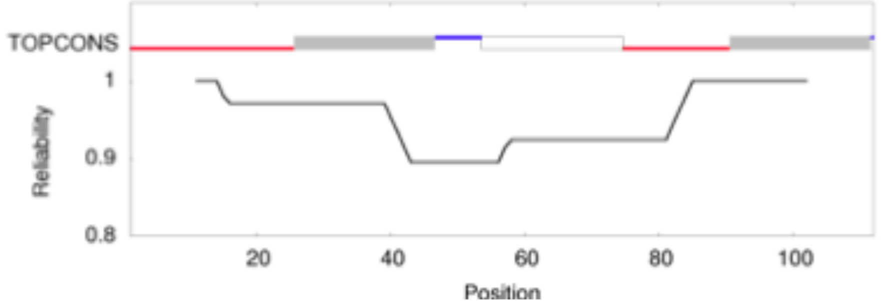

Predicted TM-helix positions:

SCAMPI-seq	1. 26-46, 2. 54-74, 3. 91-111
SCAMPI-msa	1. 25-45, 2. 54-74, 3. 91-111
PRODIV	1. 27-47, 2. 50-70, 3. 91-111
PRO	1. 25-45, 2. 50-70, 3. 91-111
OCTOPUS	1. 26-46, 2. 54-74, 3. 91-111
TOPCONS	1. 26-46, 2. 54-74, 3. 91-111

Sequence and predicted topologies:

```
                1                                                41
Seq.            MTNLTTVVQK FYDEYVNNTP KKLKLIDAYL FYIVLTGVIQ FLYCCLVGTF
SCAMPI-seq      iiiiiiiiii iiiiiiiiii iiiiiMMMMM MMMMMMMMMM MMMMMMoooo
SCAMPI-msa      iiiiiiiiii iiiiiiiiii iiiiMMMMMM MMMMMMMMMM MMMMMooooo
PRODIV          iiiiiiiiii iiiiiiiiii iiiiiiMMMM MMMMMMMMMM MMMMMMMooM
PRO             iiiiiiiiii iiiiiiiiii iiiiMMMMMM MMMMMMMMMM MMMMMooooM
OCTOPUS         iiiiiiiiii iiiiiiiiii iiiiiMMMMM MMMMMMMMMM MMMMMMoooo
TOPCONS         iiiiiiiiii iiiiiiiiii iiiiiMMMMM MMMMMMMMMM MMMMMMoooo

                51                                               91
Seq.            PFNSFLSGFI STISCFVLGV CLRLQVNPEN KQNFIGISPE RGFADFILAH
SCAMPI-seq      oooMMMMMMM MMMMMMMMMM MMMMiiiiii iiiiiiiiii MMMMMMMMMM
SCAMPI-msa      oooMMMMMMM MMMMMMMMMM MMMMiiiiii iiiiiiiiii MMMMMMMMMM
PRODIV          MMMMMMMMMM MMMMMMMMMM iiiiiiiiii iiiiiiiiii MMMMMMMMMM
PRO             MMMMMMMMMM MMMMMMMMMM iiiiiiiiii iiiiiiiiii MMMMMMMMMM
OCTOPUS         oooMMMMMMM MMMMMMMMMM MMMMiiiiii iiiiiiiiii MMMMMMMMMM
TOPCONS         oooMMMMMMM MMMMMMMMMM MMMMiiiiii iiiiiiiiii MMMMMMMMMM

                101        111
Seq.            IILHLVVFNF IG
SCAMPI-seq      MMMMMMMMMM Mo
SCAMPI-msa      MMMMMMMMMM Mo
PRODIV          MMMMMMMMMM Mo
PRO             MMMMMMMMMM Mo
OCTOPUS         MMMMMMMMMM Mo
TOPCONS         MMMMMMMMMM Mo
```
