# Supplementary figures and images for: Feeding on resistant rice leads to enhanced expression of defender against apoptotic cell death (OoDAD1) in the Asian rice gall midge
Source: BMC Plant Biol. 2015 Oct 1;15:235. doi: 10.1186/s12870-015-0618-y (PMC4591563; doi:10.1186/s12870-015-0618-y)

**A**

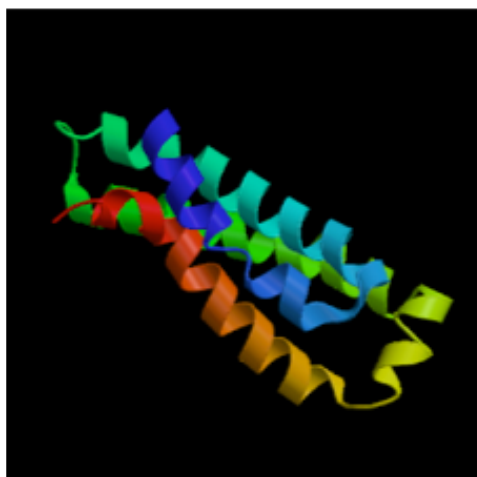

**B**

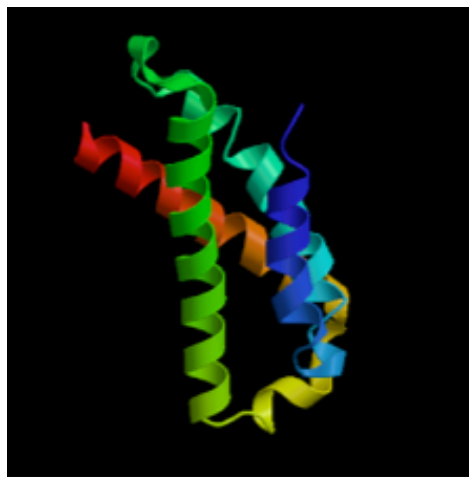

**C**

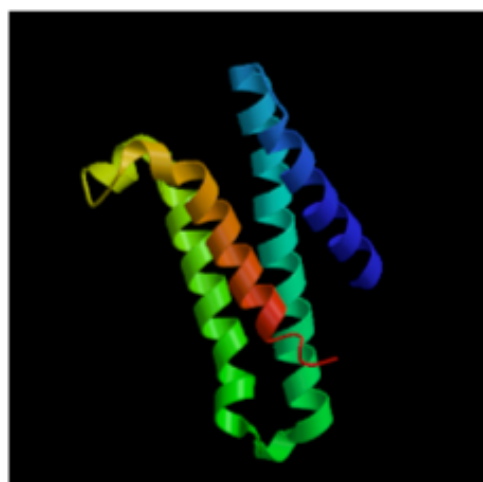

**D**

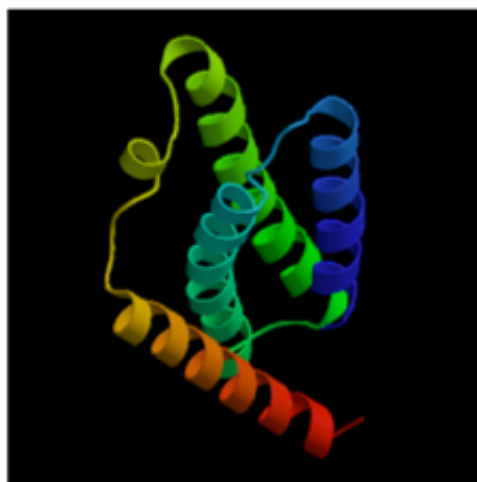

**E**

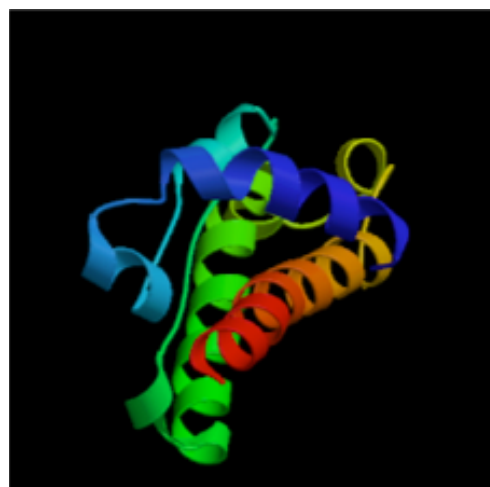

Supplement: Additional file 2: Figure S2. — Protein structure of OoDAD1 as predicted by ROBETTA, a de novo protein-threading program (http://robetta.bakerlab.org). A, B, C, D and E are the five predicted models and the fifth model was predicted to have lowest score (lowest energy) and therefore, considered the most stable. (PDF 166 kb) [file 12870_2015_618_MOESM2_ESM.pdf]

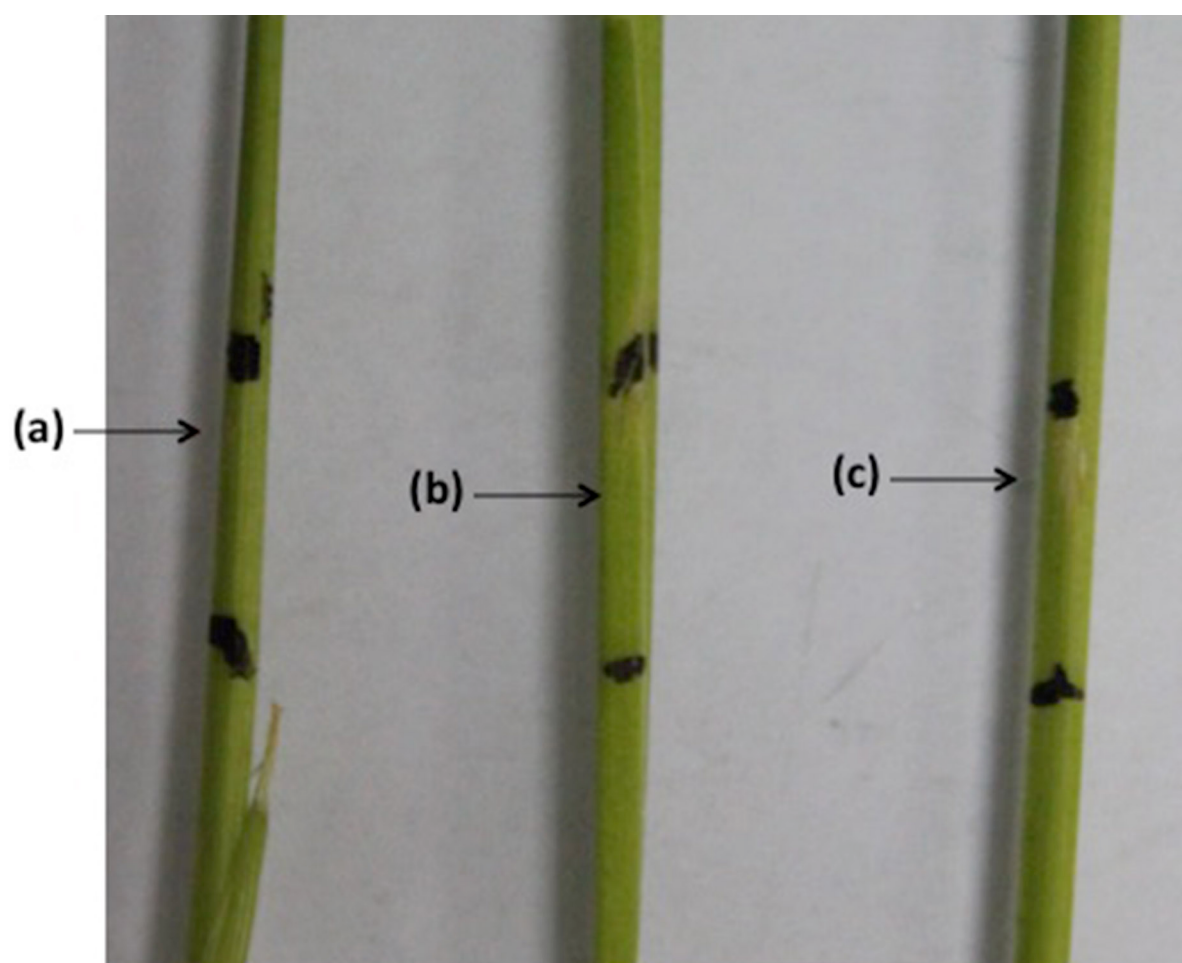

Supplement: Additional file 3: Figure S3. — HR response of gall midge resistant rice injected with recombinant OoDAD1. Plant injection assay showing induction of HR response in the gall midge resistant rice variety, Jaya, upon injection with recombinant OoDAD1. Jaya injected with (a) purified protein; (b) protein elution buffer; (c) BSA dissolved in protein elution buffer. Black dots indicate the markings made prior to injection for easy localization of injected regions. (PDF 199 kb) [file 12870_2015_618_MOESM3_ESM.pdf]
